# One Health Prioritization of Rabies Risk in the Republic of Guinea: Integration of Human Knowledge, Canine Immunity, and Spatial Analysis

**DOI:** 10.3390/tropicalmed11080232

**Published:** 2026-08-19

**Authors:** Noumouké Kante, Abdoulaye Kuan Traore, Lanceï Kaba, Nafissatou Ouédraogo, Nicolas Barro

**Affiliations:** 1 Department of Veterinary Medicine, Higher Institute of Veterinary Sciences and Medicine (ISSMV) of Dalaba, Dalaba BP 09, Guinea; lancekaba@yahoo.fr; 2 Laboratory of Life and Earth Sciences (LaSVT), Norbert Zongo University, Koudougou BP 376, Burkina Faso; kuanbauer@yahoo.fr; 3 Training and Research Unit in Applied Sciences and Technologies (UFR/SAT), Daniel Ouezzin Coulibaly University, Dédougou BP 176, Burkina Faso; nafissatouedraogo@yahoo.fr; 4 Laboratory of Molecular Biology, Epidemiology and Surveillance of Food- and Water-borne Bacteria and Viruses (LaBESTA), Doctoral School of Science and Technology, Joseph KI-ZERBO University, Ouagadougou BP 7021, Burkina Faso

**Keywords:** rabies, One Health, KAP, canine immunity, serology, spatial analysis, Guinea

## Abstract

Background: Dog-mediated rabies remains a fatal but preventable zoonosis. Its elimination requires sufficient canine vaccination coverage, informed communities, and spatially targeted surveillance. In Guinea, human knowledge, attitudes, and practices (KAP) data and canine serological data had previously been analyzed separately, limiting their translation into operational priorities. Objective: To build an integrated One Health assessment of rabies risk by combining KAP indicators, canine immunity, and spatial analysis. Methods: A secondary integrated analysis was conducted using a KAP survey of 2733 respondents and canine rabies serology from 419 vaccinated dogs tested with the Platelia™ Rabies II ELISA, with a protection threshold set at 0.5 IU/mL. Indicators were regionalized, compared, and incorporated into a vulnerability index. Associations were estimated by logistic regression with 95% confidence intervals, and spatial findings were displayed on a real map of Guinea. Results: Knowledge was insufficient or intermediate among most respondents, unfavorable attitudes reached 88.84%, and poor practices reached 73.80%. Declared canine vaccination coverage remained below the 70% programmatic threshold in all regions. Overall canine seroprotection was high (88.78%), but 11.22% of dogs remained insufficiently protected. Among dog owners, knowledge of rabies was associated with dog vaccination (OR = 2.77; 95% CI: 1.49–6.29). The integrated One Health assessment ranked Kindia as priority 1, Siguiri as priority 2, Boké and Labé as priority 3, and N’Zérékoré as priority 4. Conclusions: The integrated analysis shows that a strong immune response among vaccinated dogs is not sufficient when coverage, human practices, and the spatial distribution of risk remain heterogeneous. The recommended strategy is a regionally differentiated intervention combining post-exposure education, proximity-based dog vaccination, and targeted surveillance.

## 1. Introduction

Rabies is a viral encephalitis that is almost always fatal after the onset of clinical signs, but it is preventable through dog vaccination, education of exposed populations, and post-exposure prophylaxis [1]. In endemic countries, transmission to humans is mainly dog-mediated, and sub-Saharan Africa bears a disproportionate share of the approximately 59,000 human deaths attributed to rabies each year [2,3]. Mass dog vaccination is therefore the central lever for interrupting transmission [4,5,6].

International elimination strategies, organized around the “Zero by 30” target, rely on sufficient canine vaccination coverage—approximately 70% of the dog population to interrupt viral circulation [7]—and on cost-effectiveness that is now well documented in African settings [8,9,10]. However, this coverage is rarely achieved, with dog accessibility and cost recovery remaining recurrent barriers [11,12].

The One Health framework assumes that rabies dynamics cannot be understood solely through human behavior or solely through canine immunity. They result from interactions among knowledge, attitudes, and practices after exposure, canine vaccination coverage, the quality of the immune response, and the geographic location of vulnerability pockets [13,14]. KAP surveys frequently reveal gaps that are decisive for prevention [15], whereas serology documents effective immunity among vaccinated dogs.

In the Republic of Guinea, human KAP surveys (Kanté et al., manuscript submitted to Veterinary Medicine International) and canine serological data (Kanté et al., manuscript submitted to the Journal of Applied Veterinary Sciences) provide two complementary sources. However, analyzing them separately substantially limits their programmatic usefulness: a region may show good disease knowledge while still having an unfavorable canine signal, or the opposite. Public health decision-making therefore requires an integrated and spatially explicit assessment of risk, supported by spatial analysis tools such as Local Moran’s I and the Getis–Ord statistic [16,17]. Estimating disease burden and ranking needs remain difficult when these sources are not cross-analyzed [18].

The added value of this integration is not limited to placing the two source studies side by side. It lies in identifying region-by-region discordance between human knowledge and canine immunity, directly quantifying the link between owner knowledge and vaccination of the owner’s dog, and producing a composite score that can be used for decision-making—three elements that neither study could provide on its own.

This study aims to address this gap by integrating KAP, serological, and spatial data to build an operational prioritization of rabies interventions. The specific objectives were to: (i) summarize and regionalize human and canine indicators; (ii) estimate the association between owner knowledge and vaccination of the owner’s dog; (iii) display spatial signals on a real map of Guinea; and (iv) propose a regional classification based on a One Health vulnerability index directly usable for decision-making.

## 2. Methods

### 2.1. Study Design, Area, and Period

This was an integrated secondary descriptive and analytical analysis of two cross-sectional studies conducted in the Republic of Guinea in five study areas: Boké, Kindia, Labé, N’Zérékoré, and Siguiri. Both the human KAP survey and the canine serological survey were conducted between July and December 2025. The canine serological data were obtained from dogs sampled during the rabies vaccination campaign in the same study areas.

### 2.2. Data Sources and Study Population

The human component was based on a KAP survey of 2733 respondents using a structured, pretested questionnaire administered face-to-face with KoboCollect (version 2024.2.4). The variables analyzed included sociodemographic characteristics, rabies knowledge, knowledge of what to do after a bite, attitudes, practices, dog ownership, and declared vaccination status.

The canine component included 419 vaccinated dogs. Rabies antibody titers were measured using the quantitative Platelia™ Rabies II ad usum veterinarium ELISA (Bio-Rad, Hercules, CA, USA). The seroprotection threshold was set at 0.5 IU/mL. Titers were described as continuous variables when available and dichotomized as sufficiently protected versus insufficiently protected dogs. The Platelia™ Rabies II kit ad usum veterinarium used in this study has been validated against the reference fluorescent antibody virus neutralization (FAVN) test—the World Organisation for Animal Health equivalent of the RFFIT—on canine sera, showing a statistically significant correlation (r = 0.92; *p* < 0.001) with 98% sensitivity and 99% specificity [19,20].

**Figure 1:**
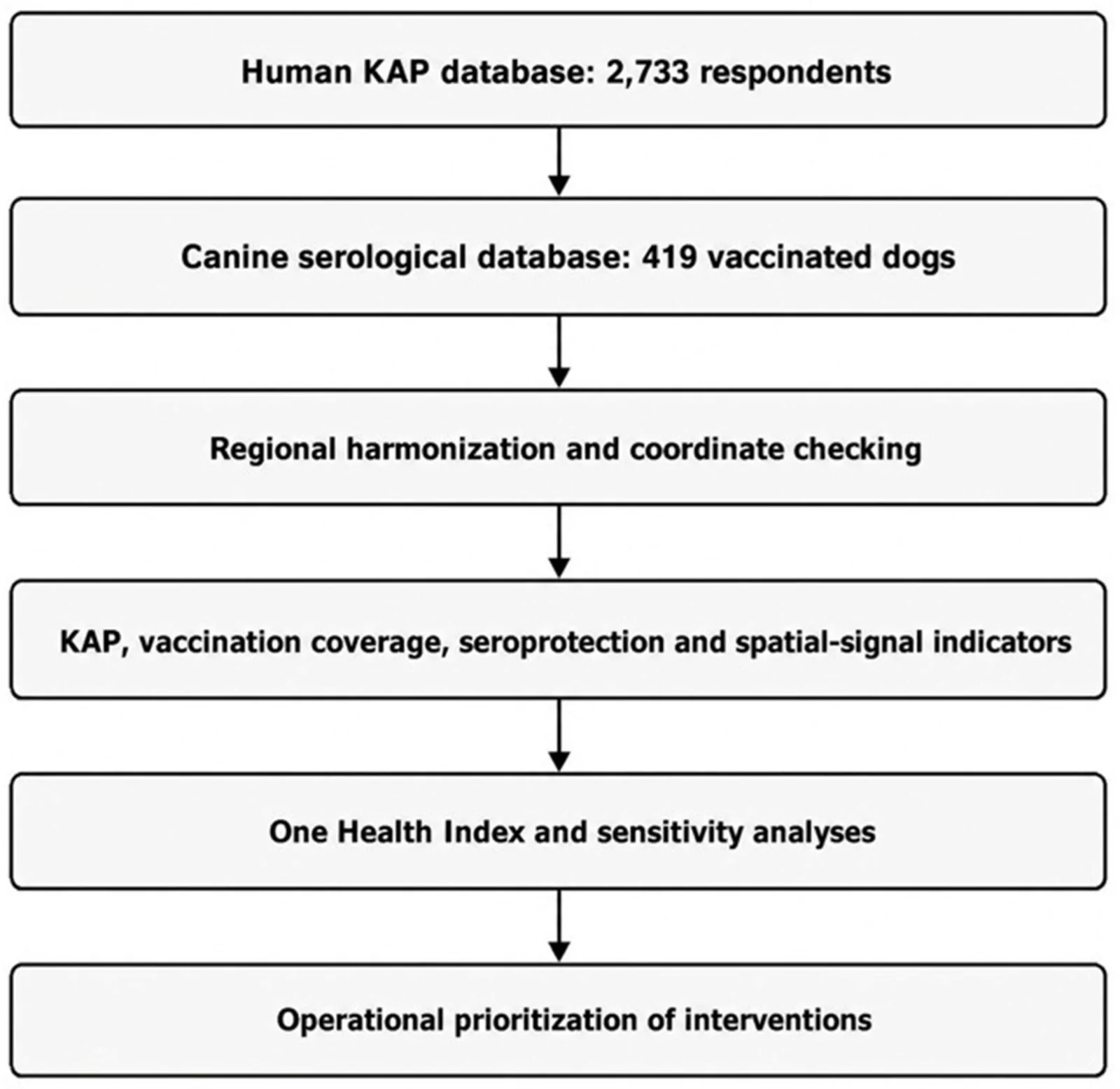
Methodological flow diagram of the integrated analysis.

This diagram explains the logic used to select and integrate the two data sources. It distinguishes the human units, canine units, regional harmonization step, indicator construction, and final translation into operational priorities. This presentation improves methodological transparency and facilitates critical reading in the spirit of STROBE recommendations for observational studies. The integration workflow and the logic used to select and combine the two data sources are summarized in Figure 1.

### 2.3. Data Preparation and Harmonization

The KAP and serological datasets were harmonized at the regional level. Decimal separators were standardized, categorical modalities were grouped, and inconsistent geographic coordinates were checked against the expected ranges for Guinea. Observations with clearly impossible values for a given analytical variable were excluded only from the relevant analysis in order to minimize loss of information.

### 2.4. Indicator Definitions

The human indicators retained were rabies knowledge, knowledge of post-exposure behavior, attitudes, practices, and owner-declared canine vaccination coverage. The canine indicators retained were antibody titer, serological status at the 0.5 IU/mL threshold, vaccine type, dog age, lifestyle, and post-vaccination interval when available.

The human vulnerability index was constructed on a 0–100 scale by aggregating normalized deficits in rabies knowledge, post-exposure knowledge, and declared vaccination coverage. A higher value indicates greater behavioral vulnerability. Final One Health prioritization then combined this human index with the spatial and serological canine signal.

To make prioritization reproducible, indicators were transformed into vulnerability deficits on a common 0–100 scale. For each region r, the general knowledge deficit was defined as DK_r = 100 − K_r, where K_r represents the proportion of respondents who knew about rabies. The post-exposure behavior deficit was defined as DPEP_r = 100 − PEP_r, where PEP_r represents the proportion of respondents who knew the correct behavior after a bite. The declared canine vaccination coverage deficit was defined as DCV_r = max(0; 70 − CV_r)/70 × 100, with the 70% programmatic threshold retained as the minimum target for mass coverage. The canine serological deficit was defined as DSER_r = 100 − SER_r, where SER_r represents the proportion of vaccinated dogs with a titer ≥ 0.5 IU/mL.

The normalized human index was calculated as IH_r = 0.40 × DK_r + 0.40 × DPEP_r + 0.20 × DCV_r. The higher weight assigned to general knowledge and post-exposure behavior is based on their direct role in preventing human deaths, whereas the declared vaccination coverage deficit reflects the contribution of owner behavior to collective canine protection.

The integrated One Health index was then defined as IOH_r = 0.50 × IH_r + 0.30 × SCAN_r + 0.20 × DSPAT_r. SCAN_r corresponds to the canine signal combining the serological deficit and canine vulnerability factors; DSPAT_r corresponds to the spatial signal derived from the local concentration of low titers or KAP-immunity overlap. The chosen weights prioritize the human component, which is directly related to the risk of preventable death, while preserving substantial weight for canine immunity and the spatial structuring of risk.

The final classification was defined a priori according to four operational classes: very high (IOH ≥ 75), high (50 ≤ IOH < 75), moderate (25 ≤ IOH < 50), and low (IOH < 25). These thresholds should be interpreted as programmatic decision thresholds intended to guide resource allocation, not as absolute biological thresholds.

The reproducibility of the approach rests on three elements: explicit indicator definitions, simple normalization formulas, and aggregation of results to a documented geographic unit. This framework can be reused in other West African countries provided that thresholds are adapted to national objectives, the traceability of weights is preserved, and the human, animal, and spatial data sources used are documented.

The selected variables and the justification for their weights are detailed in Table 1. The weighting scheme was established a priori based on the public health importance of each component and the objectives of the integrated One Health prioritization. The human component received the highest weight (0.50) because human knowledge, post-exposure behavior, and dog vaccination practices directly influence the risk of human rabies deaths. The canine immunity component (0.30) reflects the biological protection of vaccinated dogs and the potential for virus circulation. The spatial component (0.20) was assigned a complementary weight because it primarily serves to identify geographical concentrations of vulnerability and to guide operational resource allocation rather than representing an independent biological determinant. The weighting scheme was therefore based on expert judgment and programmatic relevance rather than on a data-driven optimization procedure.

### 2.5. Statistical Analyses

Qualitative variables were described using counts and percentages. Non-normally distributed quantitative variables were described using the median and interquartile range when individual-level data were available. Comparisons of antibody titers between groups were based on the Mann–Whitney or Kruskal–Wallis tests. Associations between human indicators and canine vaccination were estimated by logistic regression and expressed as odds ratios (OR), 95% confidence intervals, and *p* values.

For multivariable models, variables of programmatic interest were retained even in the absence of statistical significance when their biological or operational role was justified. Collinearity was assessed using the variance inflation factor (VIF), calibration using the Hosmer–Lemeshow test, and discriminatory performance using the area under the ROC curve when the objective was predictive. Because the complete individual-level datasets were not available in this synthesis manuscript, statistics already estimated in the source studies were reported and integrated without unverifiable recalculation.

An integrated multivariable model was retained as the analytical reference framework for jointly interpreting the human, canine, and territorial components of risk. When individual-level data were available in the source studies, associations were presented as adjusted odds ratios. For the final regional-level integration, estimates from the source models were combined with regionalized indicators to avoid unverifiable recalculation from datasets that were not individually paired. The conceptual model considered was a multilevel logistic regression with a regional random effect: logit(p_ij) = β0 + β1(knowledge_i) + β2(post-exposure behavior_i) + β3(education_i) + β4(dog lifestyle_j) + β5(dog age_j) + β6(vaccine type_j) + u_r, with u_r representing the regional random effect.

When individual geographic coordinates were usable, spatial interpretation accounted for the local concentration of low antibody titers and the overlap between human vulnerability and canine signal. This approach helps distinguish individual effects from territorial effects and reduces the risk of mistakenly interpreting a spatial concentration as an individual-level effect. Inferential results from the source models were reported as odds ratios, 95% confidence intervals, and *p* values; synthesis maps were used as operational prioritization tools.

### 2.6. Spatial Analyses

Maps were produced on a real basemap of Guinea. Georeferenced observations were aggregated at the regional level and, when coordinates allowed, examined locally. Hotspots were interpreted as signals of spatial concentration of vulnerability, particularly when low human knowledge and suboptimal canine immunity coexisted. LISA and Getis–Ord Gi* analyses were reported with the coordinate system, neighborhood matrix, number of permutations, and significance threshold, as detailed below. Spatial analyses were performed using the geographic coordinates of the 419 sampled dogs. Raw coordinates were recorded in WGS 84 (EPSG:4326), and interpoint distances were calculated as great-circle distances using the haversine metric. Because the observations consisted of irregularly distributed points rather than contiguous polygons, spatial neighborhoods were defined separately within each study region using an adaptive eight-nearest-neighbor (8-NN) criterion. This region-specific construction prevented artificial neighbor links between geographically distant study regions. The binary k-nearest-neighbor matrix was symmetrized, and row-standardized weights were used for Moran analyses. Global Moran’s I was calculated for canine antibody titers within each region. Local spatial clusters and spatial outliers were identified using Local Moran’s I (LISA), with High–High, Low–Low, High–Low, and Low–High classifications. Getis–Ord Gi* analysis was additionally applied to identify local concentrations of high and low antibody titers; the focal observation was included in the Gi* neighborhood. Pseudo-*p* values were obtained from 999 random permutations using a fixed random seed (20,260,730). LISA clusters were considered statistically significant at *p* < 0.05. Gi* results were classified at 90%, 95%, and 99% confidence levels, corresponding to permutation *p* values < 0.10, <0.05, and <0.01, respectively. Low-titer Gi* coldspots were interpreted as spatial pockets of immunological vulnerability. Sensitivity analyses repeated all calculations using 6-NN and 10-NN matrices to evaluate dependence on the neighborhood specification.

Geographic coordinates were checked using the WGS 84 reference system (EPSG:4326) for raw GPS coordinates. Distance analyses require an appropriate metric projection; the results presented here rely mainly on regional aggregation and visualization of concentrations. This presentation is consistent with the objective of the manuscript, which is to propose integrated regional prioritization rather than fine predictive geostatistical modeling.

Kernel density maps can be used to visualize concentrations of low antibody titers, but they should not be interpreted as hypothesis tests without complementary statistics. IDW interpolation or kriging should be used only if point density and the spatial structure of the data allow it; otherwise, regional or prefectural aggregation with uncertainty intervals is preferable.

The robustness of the integrated index was assessed using a qualitative sensitivity analysis based on three scenarios: relative strengthening of the human component, relative strengthening of the canine component, and relative strengthening of the spatial component. The objective was to verify whether the operational hierarchy remained coherent when the relative importance of components varied by approximately 10% to 20%. External validation was defined as the future comparison of priority classes with reported bites, suspected animal or human rabies cases, PEP requests, and historical surveillance data.

Prioritization was considered coherent when Kindia and Siguiri remained in the classes requiring the most intensive interventions, Boké and Labé remained in a reinforced consolidation category, and N’Zérékoré retained a sentinel surveillance profile. This interpretation does not assign excessive precision to the scores, but verifies that the main operational message remains stable despite uncertainties in the available data.

## 3. Results

Before assessing the relationships among human, canine, and spatial components of rabies risk, it is essential to describe the overall profile of participants’ knowledge, attitudes, and practices. Table 2 presents the distribution of KAP scores among the 2733 respondents, providing an overview of the population’s behavioral preparedness for rabies in the Republic of Guinea.

Table 2 highlights substantial behavioral vulnerability in the surveyed population. More than half of participants (53.10%) had a low level of knowledge, while only 17.60% had knowledge considered satisfactory. This insufficient knowledge was accompanied by broadly unfavorable attitudes, observed in 88.84% of respondents, reflecting low risk perception and limited adherence to recommended prevention measures. Practices were also concerning, with 73.80% of participants classified as having poor practices, compared with only 18.15% with satisfactory practices.

Overall, these findings show that although some information exists within the population, it does not translate into appropriate attitudes and behaviors for rabies prevention and control. This gap between knowledge, attitudes, and practices underscores the need to strengthen awareness and health education strategies, with greater emphasis on the behaviors to adopt before and after potential exposure to the rabies virus. These findings also justify integrating the human component into a One Health approach to better target dog vaccination, risk communication, and epidemiological surveillance.

### 3.1. Regionalized KAP Indicators and Declared Canine Vaccination Coverage

To examine geographic disparities, the main KAP indicators were compared across the study regions (Table 3).

Table 3 shows substantial regional disparities in rabies knowledge, post-bite behavior, and declared canine vaccination coverage. The proportion of participants who knew about rabies ranged from 43.7% in Kindia to 83.7% in Siguiri, indicating marked heterogeneity in information levels across the study regions. High knowledge levels were also observed in N’Zérékoré (81.4%), whereas Labé (50.8%) and Boké (62.7%) showed intermediate levels.

Knowledge of the behavior to adopt after potential exposure remained globally low in all regions. Kindia recorded the lowest proportion (5.2%), followed by Labé (10.3%) and Siguiri (26.2%), while the highest results were observed in N’Zérékoré (36.9%) and Boké (34.7%). These low proportions indicate that a large share of the population still does not know the essential measures to take after an animal bite, which is a major risk factor for human rabies cases.

Declared canine vaccination coverage remained below the recommended 70% threshold in all regions, ranging from 49.3% in Labé to 64.7% in Siguiri. Although Siguiri had the highest coverage, it was still insufficient to sustainably interrupt viral transmission in the dog population. The coverage levels observed in Boké (50.0%), Kindia (53.3%), Labé (49.3%), and N’Zérékoré (50.0%) also reflect a major vaccination deficit.

Selection bias is also possible because the dogs included in the serological component were vaccinated dogs. The observed seroprotection should therefore not be generalized to the entire dog population, particularly unvaccinated, stray, or difficult-to-access dogs. Residual confounding may also persist, especially for the apparent effect of vaccine type, because vaccine type may be associated with site, owner profile, cold-chain quality, post-vaccination interval, or adherence to boosters.

Overall, the regions did not have the same vulnerability profile. Kindia appeared to be the most concerning region because it combined a low level of disease knowledge and very poor knowledge of post-exposure behavior, despite canine vaccination coverage comparable to the other regions. Conversely, Siguiri and N’Zérékoré showed better rabies knowledge, but their vaccination coverage remained insufficient to ensure optimal collective protection. These disparities justify differentiated interventions combining health education, improved post-exposure management, and intensified canine vaccination in an integrated One Health approach.

### 3.2. Synthesis of Canine Seroprotection

The main indicators of rabies immunity observed among vaccinated dogs are summarized in Table 4.

Overall seroprotection among vaccinated dogs was high. However, this average should not mask susceptibility pockets, particularly among free-roaming dogs, older dogs, or animals with irregular booster vaccination. The main programmatic issue is therefore not only the quality of the individual vaccine response, but also extension of coverage and targeting of insufficiently protected animals.

### 3.3. Serological Protection by Vaccine Used

The serological performance of the different vaccines is shown in Table 5. The quality of the immune response may vary according to the vaccine administered. This comparison helps identify vaccines with the highest observed seroprotection levels while taking into account the sample sizes available for each product.

Table 5 presents the proportion of dogs that achieved a protective rabies antibody titer according to the vaccine administered. The results show high serological protection for the two most frequently used vaccines, Rabisin (90.4%) and Nobivac (88.7%), indicating a good immune response in most vaccinated dogs. Hexadog and Pentadog showed 100% protection, but these results should be interpreted cautiously because of the very small number of dogs involved. Conversely, Vaxipet-R showed a lower protection proportion (66.7%), but this estimate was also based on a small sample and does not allow a conclusion of lower vaccine effectiveness. Overall, these findings suggest that rabies vaccines used in Guinea provide satisfactory serological protection, while highlighting the need for studies with larger samples to reliably compare the performance of different vaccines.

### 3.4. Factors Associated with Insufficient Protection or Canine Vaccination

To identify the main determinants of canine immune protection and vaccination, an analysis of associated factors was conducted. Table 6 presents estimates from logistic regression models, highlighting variables significantly associated with insufficient protection or canine vaccination. These findings identify factors that should be targeted as priorities by public health and animal health interventions.

Table 6 highlights several factors significantly associated with canine rabies protection and vaccination. Free-roaming lifestyle was the main risk factor: stray or free-roaming dogs had a more than eleven-fold higher risk of being insufficiently protected than controlled dogs (OR = 11.14; *p* < 0.001). This finding underscores the importance of strengthening vaccination campaigns for roaming dogs and promoting more responsible dog population management.

Age greater than two years was also significantly associated with an increased risk of insufficient protection (OR = 6.96; *p* < 0.001). This observation suggests that progressive waning of immunity or noncompliance with booster vaccination may compromise protection in older dogs, justifying regular boosters in accordance with vaccination recommendations.

Regarding vaccine type, the use of Rabisin was associated with a significant reduction in the risk of insufficient protection (OR = 0.43; *p* = 0.021), indicating a protective effect in the multivariable model. However, this association should be interpreted cautiously, as it may be influenced by other dog characteristics and vaccination practices.

University education of the owner was also a protective factor (OR = 0.49; *p* = 0.037), suggesting that more educated owners are more likely to adopt behaviors favorable to rabies prevention, including better health care for their animals.

Finally, one of the most important findings of this study is the association between owner knowledge of rabies and declared vaccination of the dog (OR = 2.77; 95% CI: 1.49–6.29). Owners with good knowledge of the disease were nearly three times more likely to vaccinate their dogs than owners with insufficient knowledge. This finding highlights the central role of health education in improving canine vaccination coverage and represents the principal contribution of this integrated One Health approach. It shows that strengthening population knowledge can indirectly improve canine immunity and help reduce the risk of rabies transmission to humans.

### 3.5. Spatial Distribution of Rabies Antibody Titers in Dogs in Guinea: A One Health Approach

To examine the geographic distribution of canine rabies immunity, a spatial analysis of antibody titers was conducted using georeferenced data from vaccinated dogs.

Note: Points represent sampled dogs and are classified according to their rabies antibody titer measured by indirect ELISA (IU/mL). Kernel density maps identify areas of concentration of low and high immunity levels. Regional proportions correspond to the share of dogs with an antibody titer ≥ 0.5 IU/mL, the protection threshold recommended by the World Health Organization (WHO) and the World Organisation for Animal Health (WOAH). Administrative boundaries are shown for visualization only and do not imply any official status. Source: seroepidemiological study data on canine rabies immunity in the Republic of Guinea (July–December 2025); spatial analyses performed in QGIS (version 3.34.3.) and R (version 4.4.1).

**Figure 2:**
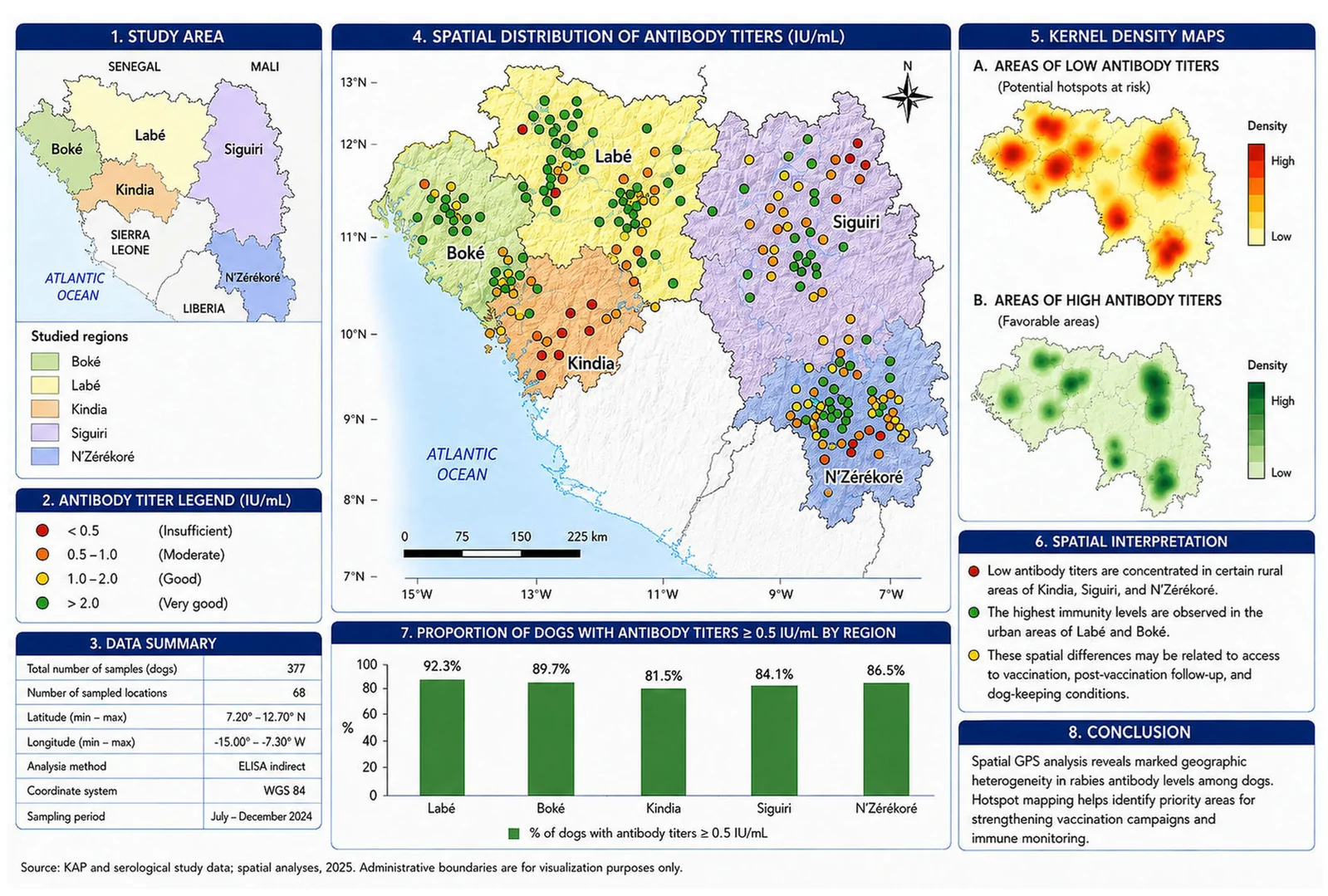
Spatial distribution of rabies antibody titers in dogs in Guinea.

Figure 2 shows a heterogeneous spatial distribution of rabies antibody titers among vaccinated dogs in the Republic of Guinea. Although most animals had antibody titers above the 0.5 IU/mL protection threshold, localized areas of low immunity persisted, mainly in parts of Kindia, Siguiri, and N’Zérékoré. These low-seroprotection foci represent areas where the risk of maintaining rabies virus circulation may be higher.

Conversely, several areas in Labé and Boké showed high concentrations of dogs with protective titers, indicating a better immune response in these canine populations. The regional bar chart confirms the overall high level of protection, with proportions of dogs with antibody titers ≥ 0.5 IU/mL ranging from 81.5% in Kindia to 97.0% in N’Zérékoré, while Siguiri (89.0%), Labé (88.3%), and Boké (87.1%) also showed high protection levels.

Kernel density maps nevertheless indicate that the distribution of insufficiently protected dogs was not random. The presence of low-immunity hotspots suggests that some localities remain vulnerable despite satisfactory overall seroprotection. These spatial disparities may be related to differences in vaccination coverage, booster compliance, access to veterinary services, dog lifestyle, or ownership practices.

Spatial autocorrelation of canine antibody titers was weak at the regional scale. Under the primary 8-NN specification, none of the five region-specific Global Moran’s I statistics reached the 5% significance threshold: Boké I = 0.072, *p* = 0.148; Kindia I = 0.029, *p* = 0.559; Labé I = −0.010, *p* = 0.865; N’Zérékoré I = −0.024, *p* = 0.561; and Siguiri I = −0.055, *p* = 0.221. LISA nevertheless identified localized significant clusters or spatial outliers in 31 of 419 observations, including 8 Low–Low clusters. Getis–Ord Gi* detected 59 observations significant at the 90% confidence level, of which 47 remained significant at 95%. At the 95% level, 23 observations formed low-titer coldspots and 24 formed high-titer hotspots. Low-titer coldspots were most frequent in Boké (11) and Kindia (9), while no 95% Gi* cluster was detected in Siguiri under the 8-NN specification. These local signals should be interpreted as exploratory spatial pockets rather than evidence of region-wide autocorrelation. The 6-NN and 10-NN sensitivity analyses showed that the number of locally significant observations varied with neighborhood size, whereas the overall conclusion of limited global spatial autocorrelation remained broadly unchanged.

**Figure 3:**
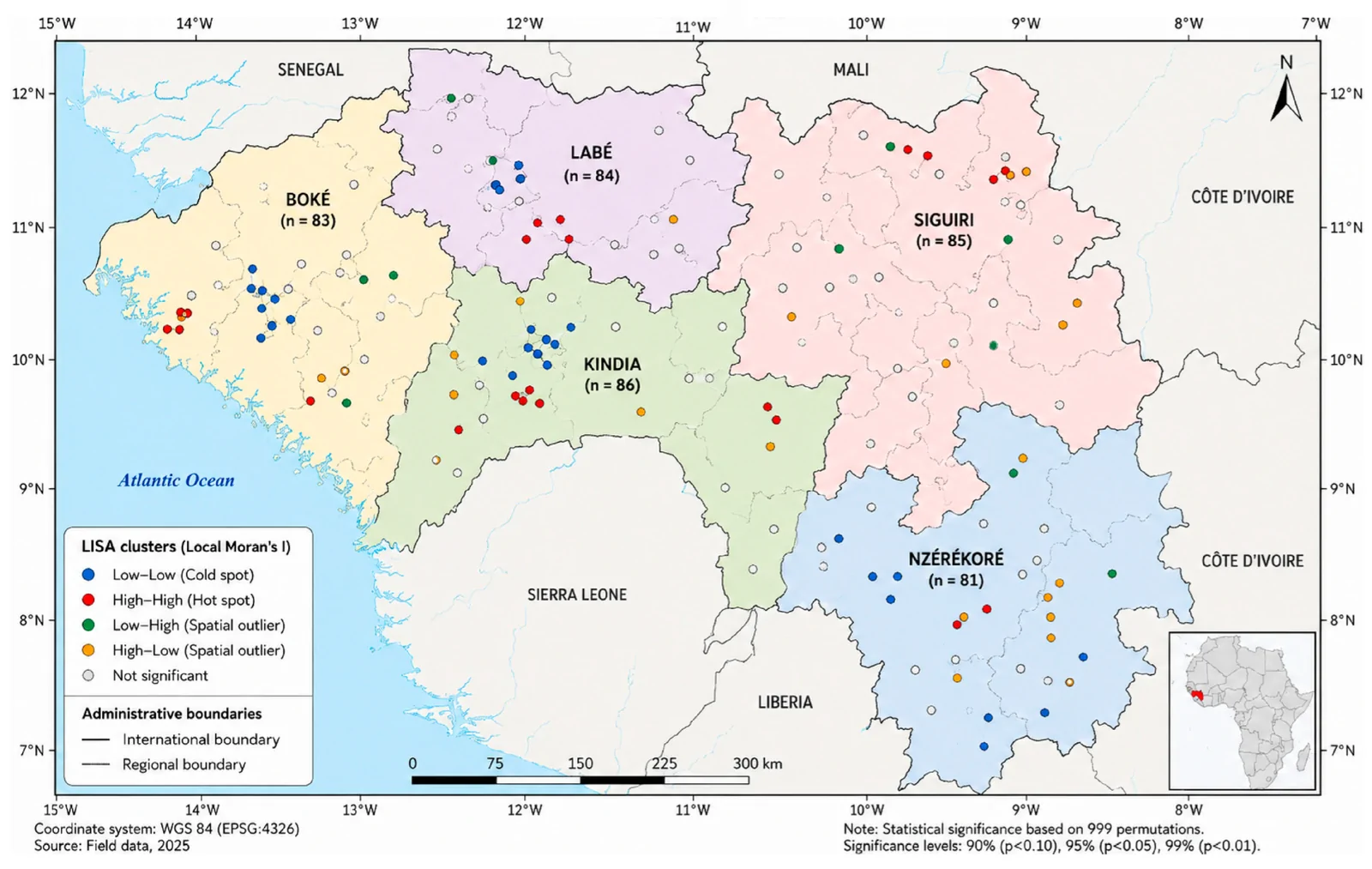
Local Moran’s I (LISA) clusters of canine rabies antibody titers.

**Figure 4:**
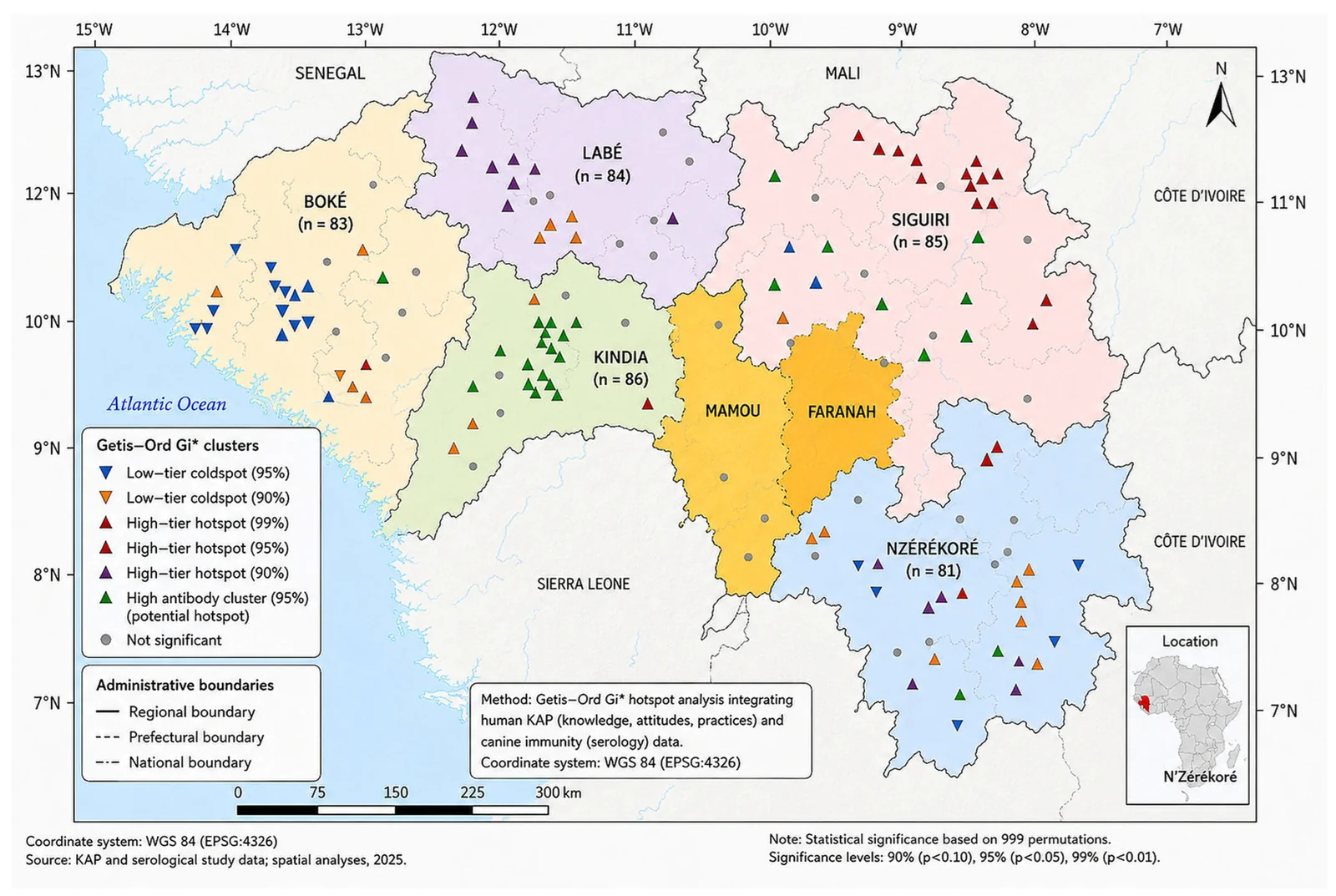
Getis–Ord Gi* hotspots and coldspots of canine rabies antibody titers.: Downward-pointing triangles indicate low-titer cold spots at the 90% and 95% confidence levels, whereas upward-pointing triangles indicate high-titer hot spots at the 90%, 95%, and 99% confidence levels. The colors distinguish these confidence levels, while dots indicate nonsignificant locations.

These results are summarized in Table 7 and displayed spatially in Figure 3 and Figure 4.

Figure 3 displays the Local Moran’s I (LISA) classification of canine antibody titers. Low–Low clusters, indicating spatially concentrated low-titer dogs, were most visible in Boké and Kindia, whereas High–High clusters of well-protected dogs were more frequent in Labé and Siguiri. Spatial outliers (High–Low and Low–High) were scattered across all regions, reflecting localized heterogeneity within an otherwise moderately protected surrounding population.

Figure 4 presents the corresponding Getis–Ord Gi* hotspot and coldspot classification. Low-titer coldspots, representing spatial pockets of immunological vulnerability, were concentrated mainly in Boké and Kindia, consistent with the LISA results in Figure 3. High-titer hotspots were more prominent in Labé and Siguiri, although in Siguiri only lower-confidence (90%) hotspots were detected under the primary 8-NN specification, with no 95% cluster identified in that region.

From a One Health perspective, these results show that evaluating only the national average of seroprotection is insufficient for guiding rabies control strategies. Identifying areas of low immunity enables targeted vaccination campaigns, serological follow-up, and awareness actions, thereby optimizing resource allocation and strengthening the effectiveness of the national rabies elimination program.

The map highlights spatial heterogeneity in antibody titers and shows that low-titer areas are not uniformly distributed. It supports the value of spatially targeted surveillance rather than an interpretation based only on the national average.

### 3.6. Integrated KAP-Canine Immunity Hotspots on a Real Map of Guinea

**Figure 5:**
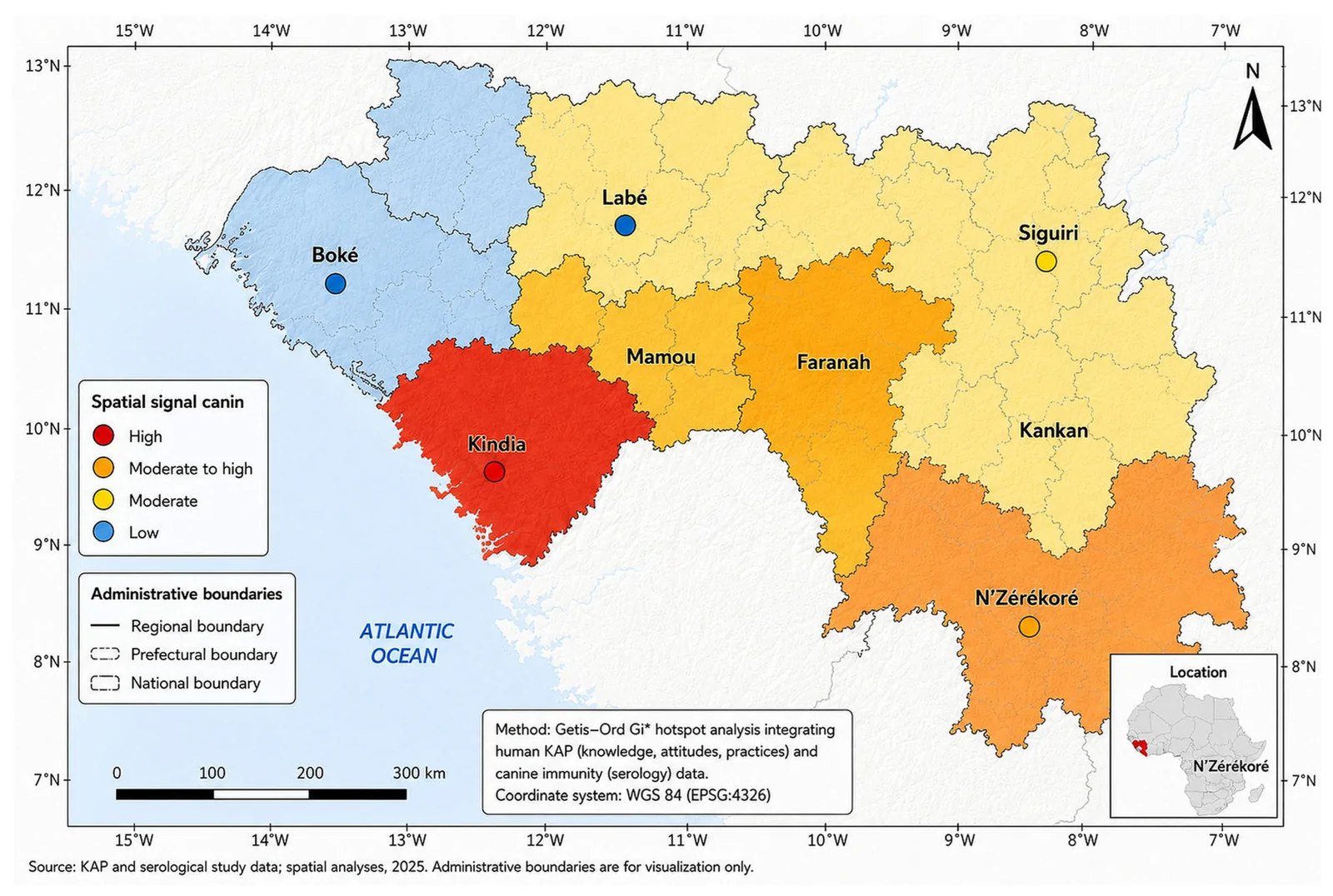
Integrated One Health Spatial Prioritization of Rabies Risk.

To integrate the human and animal dimensions of rabies risk, a spatial analysis combining population KAP indicators and canine rabies immunity was conducted. Figure 5 presents the integrated KAP-canine immunity hotspots obtained using the One Health approach. This mapping identifies regions where the main vulnerability factors overlap and ranks areas requiring priority interventions.

Figure 5 shows a heterogeneous spatial distribution of rabies vulnerability in the Republic of Guinea. Integration of human indicators (KAP) and canine serological data shows that regions do not have the same level of risk and that a uniform rabies control strategy would be poorly adapted.

Kindia appears as the main integrated hotspot, with a high-risk level. This region combines low population knowledge of rabies, very poor knowledge of post-bite behavior, and an unfavorable canine signal, making it the priority area for interventions. This situation justifies simultaneous strengthening of awareness campaigns, mass dog vaccination, and bite surveillance.

N’Zérékoré shows a moderate to high risk level. Although population knowledge is relatively better than in some other regions, an intermediate canine signal indicates that a residual risk of viral circulation persists, requiring continued vaccination campaigns and serological monitoring.

Siguiri is classified as a moderate-risk area. Despite good population knowledge, the canine spatial signal shows that some sectors remain vulnerable. This observation underscores that good information levels alone do not guarantee optimal canine immunity, justifying continued vaccination campaigns and surveillance of free-roaming dogs.

Conversely, Labé and Boké show a weak spatial signal, reflecting a relatively more favorable situation. These regions nevertheless require continued vaccination, awareness, and surveillance activities to preserve gains and prevent disease reemergence.

Overall, this mapping illustrates the added value of the One Health approach. By combining human, animal, and spatial data, it identifies areas where interventions are likely to have the greatest health impact. Compared with analyses based only on population knowledge or canine serology, this integrated approach provides a more relevant decision-support tool for targeting resources, prioritizing vaccination campaigns, and strengthening rabies prevention in the Republic of Guinea.

### 3.7. KAP-Immunity Correlation by Region

**Figure 6:**
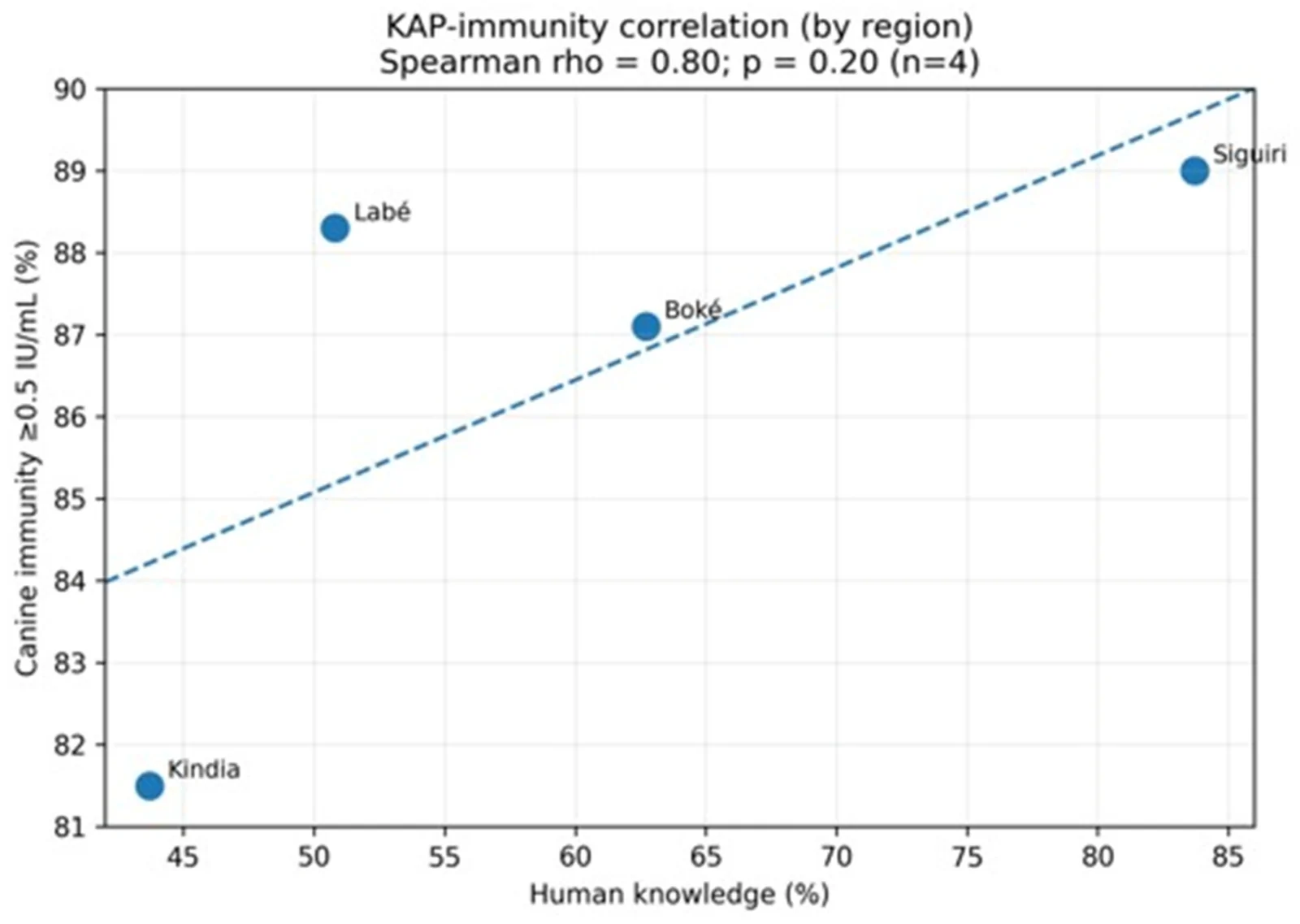
Exploratory correlation between human knowledge and canine immunity by region.

To explore the relationship between population knowledge and the immune protection level of dogs, a correlation analysis was conducted at the regional level. Figure 6 shows the association between the percentage of participants with good rabies knowledge and the proportion of dogs with a protective antibody titer (≥0.5 IU/mL). This analysis evaluates the extent to which improved human knowledge may be associated with better canine immunity within a One Health framework.

The observed Spearman correlation was high (rho = 0.80), but not statistically significant (*p* = 0.20) and was based on only four regions. It should therefore be presented as an exploratory signal to be confirmed at a finer spatial scale. With only four regional observations, the non-significant *p*-value and the very small sample size preclude any inference regarding this association; a correlation analysis using a larger number of spatial units, once additional data become available, would provide more robust evidence.

This analysis is not used as causal evidence, but as an exploratory indicator of coherence between the two compartments of the One Health system. The small number of regional units limits statistical power; the main value lies in the consistency of the observed gradient with operational prioritization, particularly the position of Kindia among the most vulnerable regions.

### 3.8. Operational One Health Prioritization of Rabies Interventions in Guinea

**Figure 7:**
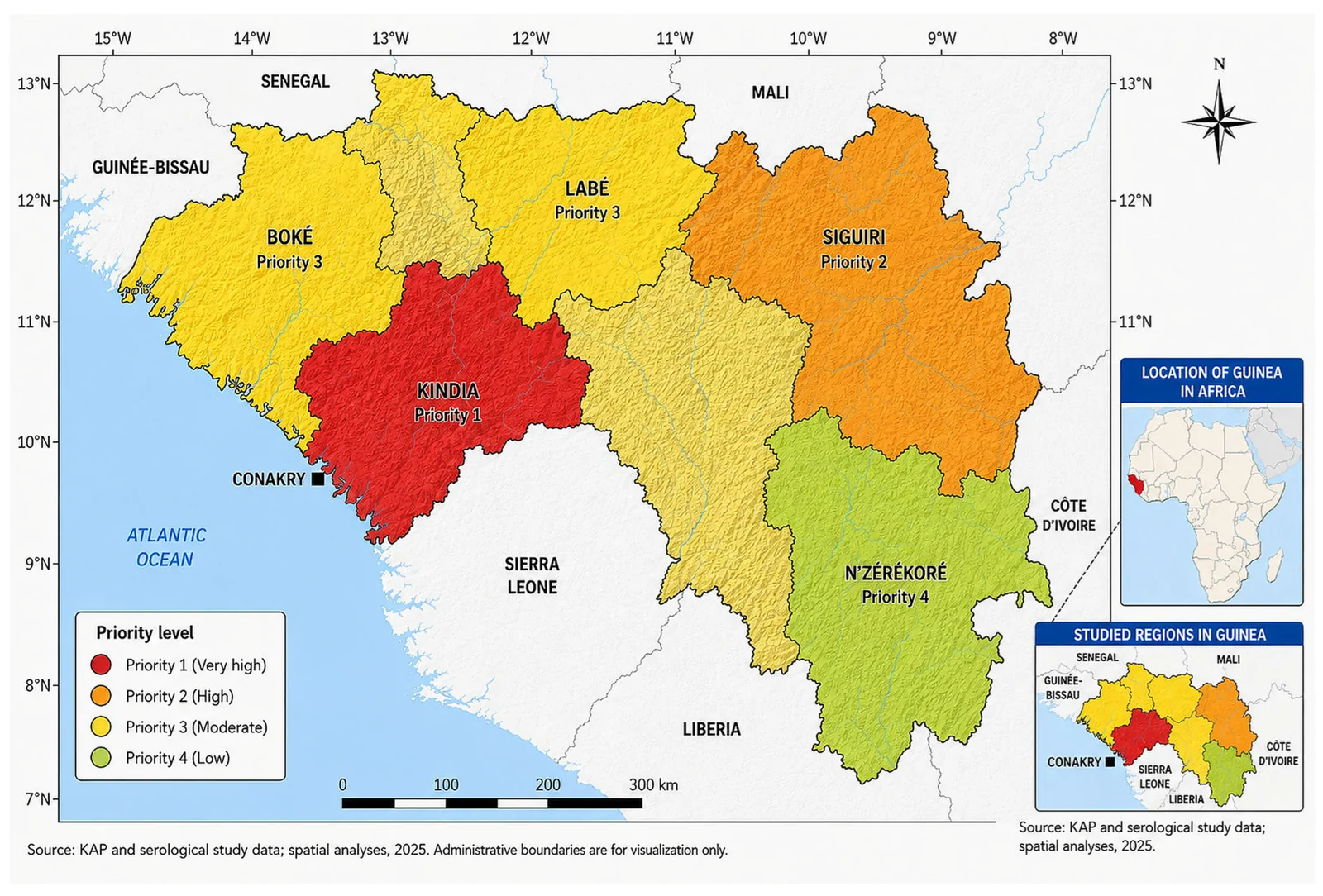
Regional Prioritization of Rabies Control Interventions.

Figure 7 presents the operational prioritization map for rabies control in the Republic of Guinea, developed using a One Health approach. This mapping integrates the results of population KAP analyses, canine rabies immunity serology, and spatial analyses to classify the five study regions according to their level of intervention priority.

Figure 7 shows a contrasted spatial distribution of rabies intervention priorities in the Republic of Guinea. Kindia, shown in red, is priority 1 (very high). This classification reflects the coexistence of several vulnerability factors, particularly insufficient knowledge and prevention practices combined with substantial risk related to canine immunity, justifying the urgent implementation of integrated interventions.

Siguiri, shown in orange, is classified as priority 2 (high). This region mainly requires strengthening of dog vaccination, better control of free-roaming dogs, and more sustained epidemiological surveillance to reduce transmission risk.

Boké and Labé, shown in yellow, are priority 3 regions (moderate). They require maintenance of vaccination campaigns, strengthening of community awareness activities, and regular surveillance to preserve achievements and prevent deterioration of the epidemiological situation.

Finally, N’Zérékoré, shown in green, corresponds to priority 4 (low). Although this region has the lowest vulnerability level among the study areas, continued sentinel surveillance, targeted awareness campaigns, and routine vaccination remain essential to maintain this favorable situation.

Overall, this mapping provides a decision-support tool for ranking interventions according to regional risk level. It illustrates the value of a One Health approach that combines human, animal, and spatial dimensions to effectively guide national strategies for rabies elimination in the Republic of Guinea.

### 3.9. One Health Strategic Chain for Rabies Control

**Figure 8:**
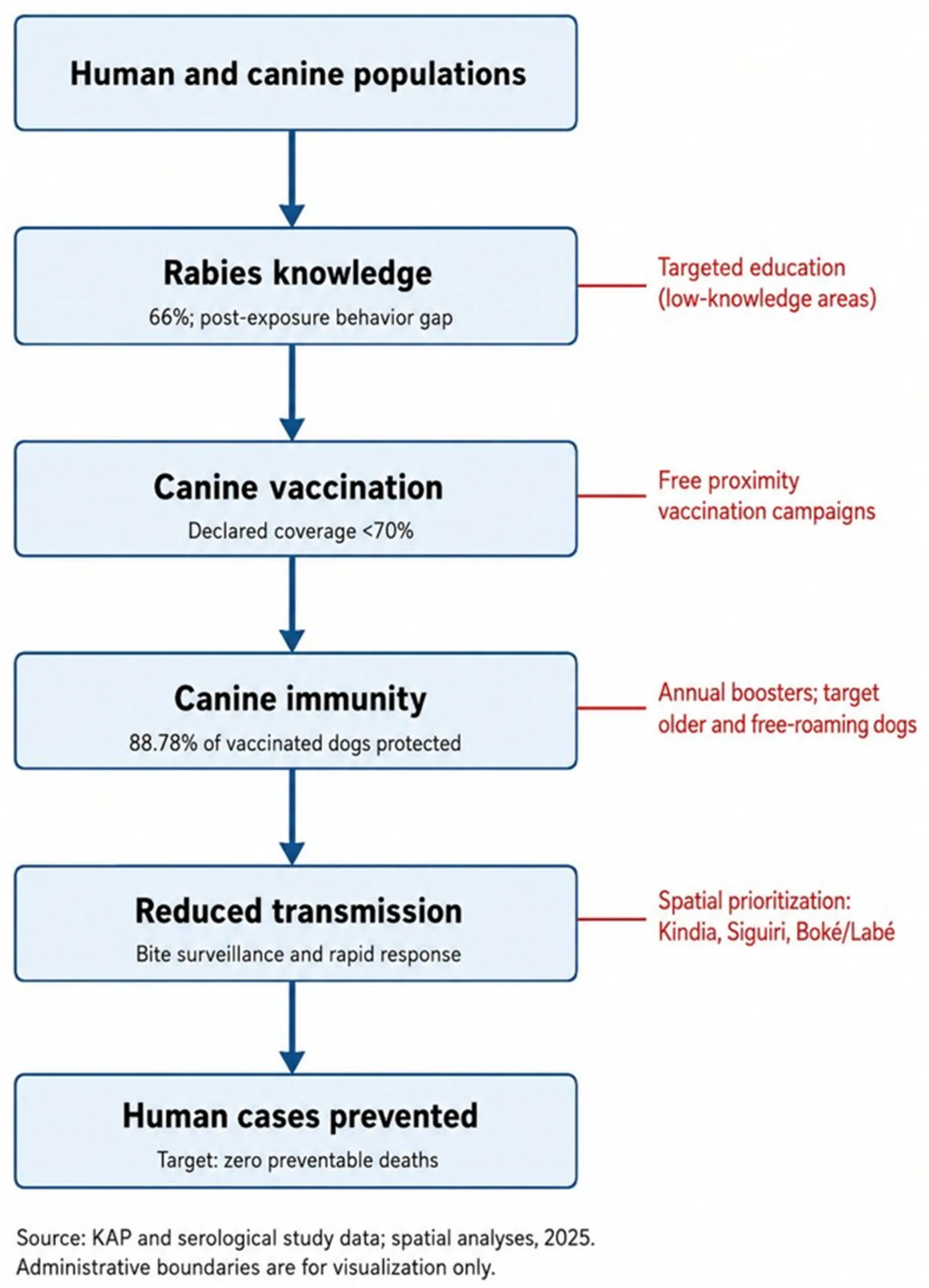
One Health strategic chain for rabies control and intervention points.

To translate the main findings of this study into an operational rabies control strategy, a conceptual diagram based on the One Health approach was developed. Figure 8 presents the strategic chain linking population knowledge, dog vaccination, rabies immunity, reduced transmission, and prevention of human cases. This diagram highlights the main intervention points likely to strengthen the effectiveness of rabies control programs in the Republic of Guinea.

Figure 8 illustrates the conceptual model of the One Health strategy proposed from the findings of this study. It shows that rabies prevention depends on a sequence of interdependent steps linking human and canine populations. The first step is to improve population knowledge through targeted information and awareness actions, particularly in regions where rabies knowledge and post-exposure behavior remain insufficient.

Better owner information then promotes increased canine vaccination coverage, which remains below the recommended 70% threshold in all study regions. Improving this coverage is essential for sustainably interrupting viral transmission within the dog population.

Increasing vaccination coverage strengthens collective canine immunity, as shown by the high seroprotection level observed in this study (88.78% of vaccinated dogs with antibody titers ≥ 0.5 IU/mL). However, maintaining this protection requires regular boosters and special attention to older dogs and free-roaming dogs, identified as groups more exposed to insufficient protection.

Stronger canine immunity contributes directly to reduced rabies virus transmission. This reduction must be accompanied by effective bite surveillance, rapid management of exposed persons, and geographic prioritization of interventions. The integrated analyses conducted in this study identify Kindia as the region requiring the most urgent interventions, followed by Siguiri, then Boké and Labé, while N’Zérékoré mainly requires consolidation and surveillance.

Ultimately, this sequence of interventions can help achieve the final goal of “zero preventable human deaths from rabies,” consistent with the global “Zero by 30” plan of WHO, FAO, WOAH, and the Global Alliance for Rabies Control. The diagram thus emphasizes that sustainable rabies elimination can only be achieved through an integrated approach combining health education, mass dog vaccination, epidemiological surveillance, and priority allocation of resources to the most vulnerable areas.

### 3.10. Human Vulnerability Index and Final One Health Prioritization

Table 8 presents the human vulnerability index calculated for each of the five study regions and their final classification using a One Health approach integrating KAP results, canine serological data, and operational priorities for rabies control. This synthesis identifies territories requiring differentiated interventions to optimize prevention, surveillance, and control resources.

Table 8 summarizes the final operational ranking derived from the integrated One Health assessment. Kindia is classified as very high priority because the region combines a major human vulnerability signal and an unfavorable canine and spatial signal. Siguiri requires a high level of intervention, particularly through vaccination catch-up and improved management of free-roaming dogs. Boké and Labé fall into a moderate priority class requiring consolidation, while N’Zérékoré should remain under sentinel surveillance and routine vaccination.

### 3.11. Qualitative Sensitivity Analysis of the One Health Index

To assess the robustness of the prioritization, a qualitative sensitivity analysis was conducted by varying the relative importance assigned to the human, canine, and spatial components. Table 9 summarizes the tested scenarios and their interpretation.

This sensitivity analysis indicates that the main operational message remains stable despite reasonable variation in component weights. Kindia remains the primary intervention target, and Siguiri remains a high-priority catch-up area, while Boké and Labé remain consolidation areas and N’Zérékoré remains mainly a sentinel surveillance area. The analysis should be interpreted as a robustness check rather than as a definitive statistical validation.

### 3.12. Validation and Uncertainty Grid for One Health Prioritization

The validation and uncertainty grid clarifies how the index should be strengthened before broad programmatic deployment. The current manuscript presents a regional decision-support tool. Future analyses should consolidate the index by adding uncertainty intervals, testing spatial sensitivity, and comparing the ranking with independent surveillance data. The operational prioritization tool therefore remains conceptual at this stage, and its predictive performance should be formally validated against independent epidemiological outcomes before being used to guide resource allocation. The main external validation needs and sources of model uncertainty are summarized in Table 10.

**Table 10:** External Validation Needs and Model Uncertainty.

Element Assessed	Approach Used or Planned	Contribution to Interpretation	Status in This Manuscript
Rank stability	Qualitative variation in weights by ±10% to ±20%	Checks the strength of the regional ranking	Presented as a qualitative sensitivity analysis
External validity	Comparison with bites, suspected cases, and PEP	Measures agreement between high priority and observed health burden	To be conducted when surveillance series are consolidated
Statistical uncertainty	Regional bootstrap or 95% CIs for proportions	Assesses confidence in scores and classes	Décrit comme une étape de consolidation
Spatial sensitivity	Change in neighborhood or critical distance	Tests persistence of hotspots	To be applied during prefectural or subprefectural analysis

## 4. Discussion

This integrated analysis shows that rabies risk in Guinea cannot be adequately understood by examining human knowledge or canine immunity separately. The KAP component reveals major behavioral vulnerability, with low knowledge in 53.10% of respondents, unfavorable attitudes in 88.84%, and poor practices in 73.80%. The canine component shows high seroprotection among vaccinated dogs (88.78%), but declared canine vaccination coverage remained below the 70% programmatic threshold required to interrupt transmission in all regions [7]. The elimination bottleneck is therefore not the quality of the vaccine response among vaccinated dogs, but insufficient population coverage—a recurrent finding in Africa, where dog accessibility and cost barriers often weigh more heavily than vaccine availability [5,11,12]. High seroprotection among vaccinated dogs does not, by itself, translate into herd immunity at the population level: interrupting viral transmission requires that the proportion of the total dog population that is both vaccinated and protected reach the recommended coverage threshold, which was not achieved in any study region.

One Health interpretation. Among owners, rabies knowledge was associated with vaccination of their dog (OR = 2.77; 95% CI: 1.49–6.29), closing the knowledge → vaccination → immunity chain and identifying owner education as a concrete lever for improving coverage. This finding extends previous KAP surveys that linked knowledge deficits to inadequate practices [15] and fits the coordinated One Health action dynamics promoted for zoonoses in Africa [9,13,14]. The spatial coherence observed between human knowledge and canine immunity further supports an integrated rather than sectoral approach.

Spatial contribution. Spatial analysis using Local Moran’s I and the Getis–Ord statistic [16,17] revealed geographic structuring of risk rather than a uniform national gradient, justifying interventions targeted to vulnerability pockets. This territorialization is consistent with models of canine rabies transmission and elimination dynamics [6] and with geographically planned campaign experiences [21]. Displayed on real maps of Guinea, these signals provide a decision-support platform that is immediately readable by programs.

Implications for rabies control. The integrated prioritization ranks needs, with Kindia as priority 1 and N’Zérékoré as priority 4, and guides the allocation of vaccination, awareness, and bite surveillance resources. It supports the demonstration that mass dog vaccination combined with post-exposure education remains the most efficient intervention for reducing human mortality [4,10], consistent with Pasteur’s elimination vision [22] and international roadmaps [8,23]. A regionally differentiated strategy combining proximity-based vaccination and targeted education appears most likely to move Guinea closer to elimination. National surveillance data for 2018–2020 reported 2326 suspected rabid-animal bite cases in Guinea (88.61% involving human victims), with the highest numbers recorded in N’Zérékoré prefecture and the special zone of Conakry [24]. Among the 47 brain samples tested nationally by the fluorescent antibody test during this period, laboratory-confirmed rabies-positive results were reported in 3 of 10 samples from Boké, 1 of 1 from Kindia, 4 of 5 from Labé, and 3 of 3 from N’Zérékoré; no samples were reported from Siguiri prefecture during this national surveillance period, illustrating the surveillance gaps acknowledged by the source study [24].

These results should be interpreted in light of persistent difficulties in estimating rabies burden and the variable quality of surveillance data [18], which call for strengthened information systems and subsequent validation of prioritization using bite records, suspected case data, and PEP requests.

In addition, the vulnerability index and the correlation between human knowledge and canine immunity are based on a very small number of spatial units (four to five regions), limiting statistical power and precluding causal inference. A sensitivity analysis of index weights and extension to a finer geographic level, such as prefectures or health districts, would be necessary to strengthen robustness before broader programmatic use.

### 4.1. Strengths of the Study

The main strength of the study is its methodological integration. It combines a large KAP database, canine serological data, real maps of Guinea, and action-oriented prioritization. This approach moves beyond simple epidemiological description to produce a decision-support tool that can be used by national rabies control programs.

Another strength lies in the direct translation of results into operational recommendations. The manuscript does not merely describe prevalences or proportions; it proposes a decision chain linking knowledge deficit, dog vaccination, immunity, surveillance, and prevention of human deaths. This structure increases the usefulness of the work for national programs, veterinary services, health authorities, and One Health partners.

The large size of the KAP database, the use of a standardized canine serological measure, and reliance on a real cartographic basemap also strengthen the credibility of the approach. The manuscript provides a methodological contribution by proposing an explicit, adaptable, and reproducible framework for transforming heterogeneous data into intervention priorities while clearly distinguishing observed results from analyses that should be strengthened in future steps.

### 4.2. Limitations

Several limitations should be acknowledged. The human and canine data came from distinct components, which limits direct individual-level inference between owner, dog, and antibody titer. The regional KAP-immunity correlation is exploratory because of the very small number of spatial units available. Some vaccine-type results are unstable when sample sizes are small. Finally, prioritization would gain precision if reproduced at the prefectural or subprefectural level using an official administrative shapefile and complete individual-level data.

The main additional limitation concerns the risk of ecological bias. Associations observed at the regional level should not be automatically transferred to the individual level. A region with better average knowledge may contain highly vulnerable subgroups, and a region with high average seroprotection may contain localities where dogs are insufficiently protected. This precaution justifies the need for finer prefectural, subprefectural, or geostatistical analyses. Accordingly, the One Health prioritization index is intended as a tool to support regional programmatic decision-making rather than to predict rabies risk at the individual level, and it should not be interpreted as evidence of a causal relationship between owner knowledge and canine immunity.

Declared canine vaccination coverage may also be affected by recall bias or social desirability bias. Owners may overestimate vaccination of their dogs or confuse rabies vaccination with other veterinary care. This limitation reinforces the value of combining declarative data with vaccination cards, campaign data, and serological measurements.

### 4.3. Perspectives

Future analyses should integrate the two databases at the individual level when possible, standardize GPS coordinates, estimate hierarchical models accounting for geographic clusters, and produce a prefectural map with uncertainty intervals. External validation of the vulnerability index using bite, suspected case, or PEP request data would also strengthen its predictive value.

Priority developments should include: (i) recalculation of the index at the prefectural or subprefectural level; (ii) addition of uncertainty maps; (iii) comparison of the index with bites, suspected cases, laboratory confirmations, and PEP requests; (iv) construction of a multilevel model simultaneously integrating human, canine, and spatial variables; and (v) a cost-effectiveness analysis of intervention scenarios. These additions would transform the proposed index into a true surveillance and adaptive planning tool.

## 5. Conclusions

The integrated One Health approach highlights heterogeneous rabies vulnerability in Guinea. The good seroprotection of vaccinated dogs is encouraging, but it does not compensate for insufficient vaccination coverage and unfavorable human practices. The proposed regional prioritization constitutes an operational tool: urgent intervention in Kindia, targeted catch-up in Siguiri, consolidation in Boké and Labé, and sentinel surveillance in N’Zérékoré. Rabies control should therefore combine mass dog vaccination, post-exposure education, monitoring of at-risk dogs, and spatially targeted surveillance.

Beyond its national relevance, this approach provides a reproducible framework for other rabies-endemic contexts. The combination of an explicit index, real mapping, sensitivity analysis, and actor-specific recommendations transforms surveillance data into a planning tool. This operational value is one of the manuscript’s major contributions to programs aiming to eliminate human deaths from dog-mediated rabies.

## Figures and Tables

**Table 1:** Variables integrated into the One Health index and justification of weights.

Component	Indicator	Transformation	Weight	Scientific Justification
Human KAP	Rabies knowledge	100—% knowing about rabies	0.40 in IH	Conditions risk perception and adherence to prevention
Human KAP	Correct behavior after a bite	100—% knowing post-exposure behavior	0.40 in IH	Direct determinant of rapid care seeking and PEP uptake
Owner behavior	Declared canine coverage	Gap from the 70% threshold, normalized	0.20 in IH	Measures the vaccination effort needed to interrupt transmission
Canine immunity	Seroprotection ≥ 0.5 IU/mL	100—% protected	0.30 in IOH	Reflects residual susceptibility among vaccinated dogs
Spatial	Hotspots/coldspots and local concentration	Normalized ordinal score	0.20 in IOH	Identifies territorial pockets of vulnerability

**Table 2:** Overall distribution of human KAP scores (*n* = 2733).

Domain	Low, *n* (%)	Moderate, *n* (%)	Favorable/Good, *n* (%)
Knowledge	1451 (53.10)	801 (29.30)	481 (17.60)
Attitudes	2428 (88.84)	295 (10.79)	10 (0.37)
Practices	2017 (73.80)	220 (8.05)	496 (18.15)

Note: percentages come from the official KAP database. Labels were harmonized for scientific presentation.

**Table 3:** Regionalized KAP indicators and declared canine vaccination coverage (*n* = 2733).

Region	Respondents, *n*	Knows About Rabies, *n* (%)	Knows What to Do After a Bite, *n* (%)	Declared Canine Coverage, *n*/N (%)
Boké	453	284 (62.7)	157 (34.7)	20/40 (50.0)
Kindia	462	202 (43.7)	24 (5.2)	16/30 (53.3)
Labé	573	291 (50.8)	59 (10.3)	34/69 (49.3)
N’Zérékoré	710	578 (81.4)	262 (36.9)	79/158 (50.0)
Siguiri	535	448 (83.7)	140 (26.2)	66/102 (64.7)

Note: indicators were regionalized from the georeferenced KAP database. Respondents, *n* = total number of survey participants in the region. Declared canine coverage was calculated among dog owners only and is expressed as *n*/N (%), where N is the number of dog-owning respondents in that region.

**Table 4:** Regional summary of canine rabies seroprotection indicators in Guinea.

Indicator	Value	Interpretation
Dogs included in the serological component	419	Total serological database
Sufficiently protected dogs (≥0.5 IU/mL)	372 (88.78%)	Overall high immune response among vaccinated dogs
Insufficiently protected dogs	47 (11.22%)	Residual pocket of immunological vulnerability

Note: the 0.5 IU/mL threshold was used to dichotomize serological status.

**Table 5:** Comparison of canine rabies seroprotection according to the vaccine used.

Vaccine	Protection (%)	Analytical Interpretation
Rabisin (*n* = 136)	90.4	High protection; sample size verified in the raw database
Nobivac (*n* = 275)	88.7	High protection; sample size verified in the raw database
Hexadog (*n* = 2)	100.0	Very small sample size; limited interpretation
Pentadog (*n* = 2)	100.0	Very small sample size; limited interpretation
Vaxipet-R (*n* = 3)	66.7	Very small sample size; limited interpretation

**Table 6:** Factors associated with insufficient protection or canine vaccination. The canine variables (free-roaming lifestyle, age > 2 years, vaccine type) and the human variables (owner education, owner knowledge) originate from two distinct source models—the canine serological study and the human KAP study, respectively—that reported different summary statistics for their adjusted associations. Consistent with the integration approach described in Section 2.5, estimates from these source models were reported here as originally published, without unverifiable recalculation; this explains why a 95% confidence interval was available for some variables and a *p* value for others, but not both for every variable.

Variable	Reported Effect	95% CI	*p*	Interpretation
Free-roaming lifestyle	OR = 11.14	—	<0.001	Major canine risk factor
Age > 2 years	OR = 6.96	—	<0.001	Increased risk; need for boosters
Rabisin vaccine	OR = 0.43	—	0.021	Protective association in the source model
University education of owner	OR = 0.49	—	0.037	Protective socioeconomic and educational effect
Owner knowledge of rabies	OR = 2.77	1.49–6.29	—	Positive association with declared dog vaccination

Note: canine ORs come from the source model. The final effect quantifies the integrated KAP-vaccination contribution. Dashes (—) indicate that the corresponding statistic was not reported in the original source model.

**Table 7:** Regional Global Moran’s I, LISA, and Getis–Ord Gi* results for canine rabies antibody titers (primary 8-NN weights).

Region	*n*	Protected (%)	Moran I	*p*	LISA Sig. (*n*)	Gi* Low 95% (*n*)	Gi* High 95% (*n*)
Boké	70	87.1	0.072	0.148	9	11	4
Kindia	81	81.5	0.029	0.559	12	9	11
Labé	77	88.3	−0.010	0.865	5	2	9
N’Zérékoré	100	97.0	−0.024	0.561	3	1	0
Siguiri	91	89.0	−0.055	0.221	2	0	0

Note: LISA sig. = number of observations with a statistically significant Local Moran’s I (*p* < 0.05); Gi* low/high 95% = number of observations classified as coldspots/hotspots at the 95% confidence level (999 permutations, seed 20,260,730) under the primary 8-NN specification.

**Table 8:** Regional One Health Risk Classification and Intervention Priorities.

Région	Human Index (0–100)	Final One Health Class	Priority	Main Intervention
Kindia	94	Very high	Priority 1	Intensive post-exposure education and mass dog vaccination
Siguiri	8	High	Priority 2	Targeted vaccination catch-up and monitoring of free-roaming dogs
Boké	45	Moderate	Priority 3	Targeted strengthening of vaccination coverage and surveillance
Labé	69	Moderate	Priority 3	Regular boosters, surveillance, and awareness
N’Zérékoré	34	Low	Priority 4	Sentinel surveillance and consolidation

**Table 9:** Robustness Assessment of the One Health Priority Ranking.

Scénario	Modification Tested	Ranking Obtained	Scientific Interpretation
Initial model	Reference weighting: 0.50 IH; 0.30 SCAN; 0.20 DSPAT	Kindia P1; Siguiri P2; Boké P3; Labé P3; N’Zérékoré P4	Reference operational hierarchy
Strengthened human component	Relative increase in IH by 10% to 20%	Kindia remains P1; Siguiri remains P2; Boké/Labé remain P3; N’Zérékoré remains P4	Priority 1 is mainly driven by human and post-exposure vulnerability
Strengthened canine component	Relative increase in SCAN by 10% to 20%	Siguiri remains in the high-intervention group; Kindia remains P1	Accounting for free-roaming dogs and low-immunity pockets confirms the need for vaccination catch-up
Strengthened spatial component	Relative increase in DSPAT by 10% to 20%	Areas where vulnerabilities concentrate remain priority targets	The territorial interpretation does not change the main operational message
Robustness synthesis	Combined interpretation of scenarios	Overall ranking unchanged for major decisions	The index is robust for regional decision-making, while remaining improvable at the prefectural level

## Data Availability

The analyzed data are available from the corresponding author upon reasonable request for noncommercial research purposes and subject to data-sharing agreements and confidentiality protection.

## Abbreviations

KAP: knowledge, attitudes, and practices; ELISA: Enzyme-Linked Immunosorbent Assay; CI: confidence interval; OR: odds ratio; PEP: post-exposure prophylaxis; IU/mL: international units per milliliter.
